# First Reports and Morphological and Molecular Characterization of *Pratylenchus delattrei* and *Quinisulcius capitatus* Associated with Chickpea in Ethiopia

**DOI:** 10.2478/jofnem-2023-0027

**Published:** 2023-06-11

**Authors:** Habtamu Kefelegn, Beira Hailu Meressa, Sunheng Yon, Marjolein Couvreur, Wim M. L. Wesemael, Misghina G. Teklu, Wim Bert

**Affiliations:** Nematology Research Unit, Department of Biology, Ghent University, Campus Ledeganck, Ledeganckstraat 35, B-9000 Ghent, Belgium; College of Agriculture and Veterinary Medicine, Jimma University, P.O. Box, 307, Jimma, Ethiopia; Flanders Research Institute for Agriculture, Fisheries and Food (ILVO), Burg Van Gansberghelaan 96, B-9820 Merelbeke, Belgium; Laboratory for Agrozoology, Department of Plants and Crops, Ghent University, Coupure links 653, B-9000 Ghent, Belgium; Plant Research, Plant Sciences Group, Wageningen University and Research Centre, P.O. Box 16, 6700 AA Wageningen, The Netherlands.

**Keywords:** D2-D3 of 28S, ITS, *COI*, *Cicer arietinum*, Ethiopia, Lesion nematode, Morphometrics, Morphology, Phylogeny, Plant-parasitic nematodes, SEM, Stunt nematode

## Abstract

Chickpea (*Cicer arietinum* L.) is classed among the most important leguminous crops of high economic value in Ethiopia. Two plant-parasitic nematode species, *Pratylenchus delattrei* and *Quinisulcius capitatus*, were recovered from chickpea-growing areas in Ethiopia and characterized using molecular and morphological data, including the first scanning electron microscopy data for *P. delattrei*. New sequences of D2-D3 of 28S, ITS rDNA and mtDNA *COI* genes have been obtained from these species, providing the first *COI* sequences for *P. delattrei* and *Q. capitatus,* with both species being found for the first time on chickpea in Ethiopia. Furthermore, *Pratylenchus delattrei* was recovered in Ethiopia for the first time. The information obtained about these nematodes will be crucial to developing effective nematode management plans for future chickpea production.

Chickpea (*Cicer arietinum* L.) is classed second among the most important leguminous grain crops after the common bean, and is grown throughout tropical, subtropical, and temperate regions (Singh et al., 2008; FAOSTAT, 2020). Ethiopia is the largest producer of chickpeas in Africa, contributing 60% of the continent's total production and ranking sixth internationally (Shiferaw et al., 2007; FAOSTAT, 2020; Fikre et al., 2020). Chickpea is grown in Ethiopia for both domestic consumption and export purposes. It is also used to restore soil fertility as part of a crop rotation with wheat and teff (Dadi et al., 2005; Shiferaw et al., 2007; Fikre et al., 2020).

In Ethiopia, growers of chickpea experience different diseases and insect pests in their fields for which management methods are being implemented, and although plant-parasitic nematodes also represent an important chickpea pest, their importance is usually neglected due to local inabilities to recognize relevant symptoms and/or in identifying the associated species (Castillo et al., 2008; Abebe et al., 2015; Sikora et al., 2018). The root-lesion nematodes (RLN), *Pratylenchus* spp., are ranked as the third most damaging group of plant-parasitic nematodes in terms of economic loss to agricultural production after root-knot and cyst nematodes (Castillo and Vovlas, 2007; Jones et al., 2013). *Pratylenchus* is the most important genus that infects chickpea roots globally and reduces crop yields (Di Vito et al., 1992; Thompson et al., 2010; Reen et al., 2019; Behmand et al., 2022; Rostad et al., 2022), and various *Pratylenchus* species from chickpea roots and rhizospheres have been reported from countries in Asia, Africa, Europe, North America, South America, and Australia (Castillo et al., 2008; Sikora et al., 2018; Zwart et al., 2019). According to studies by Hollaway et al. (2000) and Behmand et al. (2018), in different parts of Turkey where chickpea is grown, chickpea crops are generally considered as being more susceptible to *P. neglectus*, *P. penetrans*, and *P. thornei* attack than field pea, fava bean, and lupin bean crops, but less vulnerable than wheat crops. In Australia, *P. thornei* and *P. neglectus* are known to cause substantial damage to chickpea production (Riley and Kelly, 2002; Hollaway et al., 2008; Thompson et al., 2010). Likewise, *P. thornei* has been reported to cause severe crop losses in Syria, Morocco, Tunisia, Algeria, India, and Spain (Di Vito et al., 1992; Di Vito et al., 1994; Castillo et al., 1996; Ali and Sharma, 2003).

The stunt nematode, *Quinisulcius capitatus* (Allen, 1955) Siddiqi, 1971 (= *Tylenchorhynchus capitatus* Allen, 1955) is a polyphagous ectoparasite with a wide host range, common in leguminous crops (Greco et al., 1992), field peas in the USA (Upadhaya et al., 2018), and is commonly found parasitizing chickpea fields in Tunisia, Morocco, and Turkey (Di Vito et al., 1994; Ali and Sharma, 2003; Catillo et al., 2008). *Quinisulcius* species are also widely distributed throughout tomato, pepper, cabbage, and potato crops in many countries worldwide (Bafokuzara, 1996; Baimey et al., 2009; Geraert, 2011; Hussain et al., 2019). The correct identification of nematodes using the link between DNA sequences and morphological characters is crucial in avoiding species misidentification (Janssen et al., 2017a, 2017b), and therefore for the implementation of effective pest management strategies and control measures (Munawar et al., 2021). Nevertheless, in sub-Saharan Africa (SSA), where facilities for morphological and molecular characterizations are scarce, nematode identification has hitherto been limited to genus level (Powers et al., 2011; Coye et al., 2018). For example, in Ethiopia, despite the number of described species of *Pratylenchus* (Janssen et al., 2017b; Singh et al., 2018; Nguyen et al., 2019; Handoo et al., 2021) and *Quinisulcius* (Geraert, 2011; Hussain et al., 2019), only *P. goodeyi* from enset (Peregrine and Bridge, 1992) and *P. zeae*, *P. brachyurus*, and *P. coffeae* from maize have been identified to date (Abebe et al., 2015). This current study reports for the first time the presence of *P. delattrei* in Ethiopia, and in addition, it provides the first report of *P. delattrei* and *Q. capitatus* associated with chickpea. This study also characterizes these two species based on morphological features obtained from light microscope (LM) and scanning electron microscope (SEM), molecular information of ITS, 28S of rDNA and *COI* of mtDNA. Overall, the study provides a better understanding of nematodes as a potential concern in chickpea production in the country.

## Materials and Methods

### Sample collection and nematode extraction

Soil and root samples were collected from chickpea growing areas in Minjar, Adea’a, and Mesekan districts during the 2021 main growing season, located in central and southern parts of Ethiopia. Details regarding sample locality, altitude, GPS coordinators, and GenBank accession numbers are summarized in Table 1. From each sampling locality, 20 soil cores were taken in a zig-zag pattern from within the top 30 cm using a 3 cm diameter tube from the chickpea rhizosphere, mixed to obtain a 500 g soil sample. For each sample, 80 chickpea roots were collected and put in labelled plastic bags. Subsequently, both soil and root samples were taken to the Plant Disease Diagnostics laboratory at Jimma University and stored at 4°C until nematode extraction (Barker et al., 1969). The nematodes were extracted from aliquots of 100 ml of soil and 10 g of roots by the modified Baermann tray method described by Hooper et al. (2005).

**Table 1.** *Pratylenchus delattrei* and *Quinisulcius capitatus* recovered from chickpea roots and soil, districts, sampling locality, laboratory codes, altitude, GPS coordinators and GenBank accession numbers.

**Districts**	**Sampling locality**	**Codes**	**Species**	**Altitude (m)**	**Longitude(°)**	**Latitude(°)**	**GenBank accession number**		

							**28S–rDNA**	**ITS–rDNA**	***COI* gene**
Minjar	Kitecha	Mki-5	*P. delattrei*	1600-1800	8°52′58.16″N	39°29′46.01″E	OP646170	-	-
		Mki-8					OP646169	-	-
		Mki-12					-	-	OP730534
Adea’a	Gollodhertu	AG-1	*P. delattrei*	1800-1900	8°38′55.54″N	38°55′0.94″E	-	OP646171	-
		AG-2					OP646168	OP646172	-
		AG-3					OP646167	-	OP730535
Mesekan	Jolle-2	JO2-3	*Q. capitatus*	1900-1950	8°11′49.79″N	38°27′51.24″E	OP626319	OP646173	OP627909
		JO2-5					OP626320	OP646174	OP627910
		JO2-7					OP626321	OP646175	OP627911

### Morphological characterization

Morphological and morphometric data were recorded from both temporary and permanent slides. In order to link molecular data with morphological vouchers of individual nematodes, live nematodes were heat relaxed by quickly passing them over a flame and examined, photographed, and measured using an Olympus BX51 DIC Microscope (Olympus Optical, Tokyo, Japan), equipped with an HD Ultra camera. Subsequently, each specimen was recovered from the temporary slide for genomic DNA extraction. For permanent slides, the nematode suspensions were concentrated in a drop of water in an embryo glass dish, with a few drops of fixative (4% formalin, 1% glycerol (in water) in it. The nematodes were immediately heated in a microwave (700 watts) for about 4 sec and left at room temperature for 1 h at 4°C for 24 h. This was followed by gradually transferring to anhydrous glycerin, ready to be mounted on glass slides as described by Seinhorst (1959). Specimens for scanning electron microscopy (SEM) were fixed in Trump's fixative, washed in 0.1 M-phosphate buffer (pH = 7.5), dehydrated in a graded series of ethanol solutions, critical point dried with liquid CO_2_ and mounted on stubs with carbon tabs (double conductive tapes), coated with 25 nm gold, and photographed with a JSM-840 EM (JEOL) at 12 kV (Singh et al., 2021).

### Molecular characterization

After making morphological vouchers, nematodes were recovered from temporary slides, washed with distilled water, cut into 2–3 pieces, and transferred to a PCR tube containing 20 μL of worm lysis buffer (WLB) (50 mM KCl;10 mM Tris pH 8.3; 2.5 mM MgCl2; 0.45% NP-40 (Tergitol Sigma); 0.45% Tween-20). Then, the samples were incubated at −20°C for 10 min, followed by adding 1 μL proteinase K (1.2 mg/ml) and incubation for 1 h at 65°C and 10 min at 95°C and centrifugation for 1 min at 14,000 rpm. Finally, the samples were stored at −20°C until used for the PCR, as previously described by Singh et al. (2019) and Nguyen et al. (2019). A DNA template of 3 μL was transferred to an Eppendorf tube containing 23.5 μL master mix containing 10 μL of PCR water, 12.5 μL Dream tag, and 0.5 μL of each primer (Derycke et al., 2010) and PCR amplification was performed using a Bio-Rad T100™ thermocycler. PCR amplifications of the D2-D3 region of 28S-rDNA were performed using the forward primer D2A (5′-ACA AGT ACC GTG AGG GAA AGT TG-3′), and reverse primer D3B (5′-TCG GAA GGA ACC AGC TAC TA-3′) (Subbotin et al., 2006). For ITS rDNA, the forward primer Vrain2F (5′-CTT TGT ACA CAC CGC CCG TCG CT-3′), and reverse primer Vrain2R (5′-TTT CAC TCG CCG TTA CTA AGG GAA TC-3′), were used following the protocol of Vrain et al. (1992) with the touch-down thermal profiles described by Singh et al. (2019). For the amplification of the cytochrome oxidase subunit 1 (*COI*) gene of mitochondrial DNA, the primer JB3 (5′-TTT TTT GGG CAT CCT GAA GTC TAT-3′) and JB4.5 (5′-CCT ATT CTT AAA ACA TAA TGA AAA TG-3′) and the primer JB3Prat (5′-TTT TTT GGG CAT CCT GAA GTC TAT-3′) and JB4Prat (5′-CCT ATT CTT AAA ACA TAA TGA AAA TG-3′) were used following the protocol of Bowles et al. (1992) with the thermal profile described in the study of Singh et al. (2019). All the PCR products were checked by gel electrophoresis stained with GelRed (Biotium) and visualized under UV light illumination. The successful PCR reactions were purified and sent to Macrogen (https://dna.macrogen.com, Europe) for sequencing. Consensus sequences were assembled in forward and reverse directions using Geneious 2022.1 (Biomatters; http://www.geneious.com) and deposited in the NCBI GenBank (Table 1).

### Phylogenetic analysis

Resulting sequences were compared with other relevant sequences available in the GenBank. Multiple alignments of the different DNA sequences were made using MUSCLE with default parameters, followed by manual trimming of the poorly aligned ends using Geneious 2022.1. Phylogenetic trees were created by using MrBayes 3.2.6, adding Geneious with the GTR + I + G model. The Markov chains for generating phylogenetic trees were set at 1 × 106 generations, four runs, 20% burn-in and sub-sampling frequency of 500 generations (Huelsenbeck and Ronquist, 2001).

## Results

*Pratylenchus delattrei* Luc, 1958

(Fig. 1).

**Figure 1:**
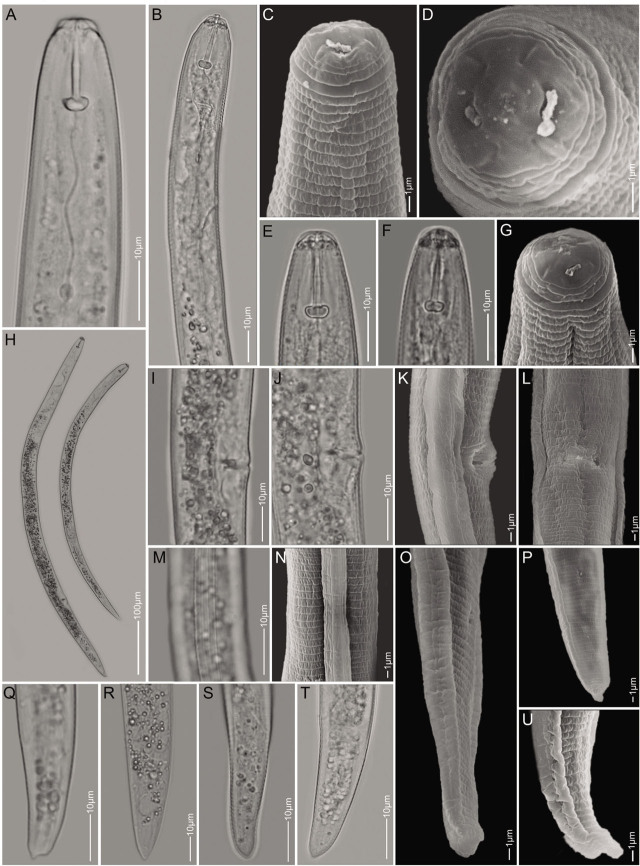
Light microscopy and scanning electron microscopy images of *Pratylenchus delattrei*. A–C, E–G: Anterior part of the body showing lip and neck region; D: *En face* view; H: Whole female's body; I–L: Vulva region (L, ventral view); M,N: Lateral fields at mid-body; O–U: Tail region.

### Measurements

See Table 2.

**Table 2.** Comparison of morphometrics of the Ethiopian *Pratylenchus delattrei* from chickpea in Ethiopia with the original description from Madagascar (Luc, 1958), and two other *P. delattrei* populations from Cape Verde and Iran. All measurements are in μm and in the form: mean ± s.d. (range).

**Character**	***P. delattrei* from chickpea in Ethiopia (present study)**	***P. delattrei* from cotton in Madagascar (Luc, 1958)**	***P. delattrei* from tomato in Cape Verde (Flis et al., 2018)**	***P. delattrei* from vegetables in Iran (Majd Taheri et al., 2013)**	

				**Hormozgan 1**	**Hormozgan 2**
n	10	13	20	7	12
L	475 ± 47.1 (410 – 560)	390 – 470	532 ± 33 (498 – 586)	543 ± 55 (467 – 616)	508 ± 49.2 (434 – 576)
a	25.2 ± 2.2 (22.8 – 29)	20.4 – 25.8	26.6 ± 2.2 (22.1 – 31.3)	23.8 ± 2.1 (21.2 – 26.9)	22.6 ± 1.0 (21.1 – 25)
b	5.6 ± 0.6 (4.8 – 6.6)	3.7 – 4.8	6.6 ± 0.5 (6.1 – 7.7)	6.1 ± 0.7 (5.0 – 7.2)	5.9 ± 0.6 (5.2 – 6.9)
b’	4.7 ± 0.5 (4.1 – 5.6)	–	4.5 ± 0.4 (4.0 – 5.3)	4.3 ± 0.2 (4.0 – 4.6)	4.1 ± 0.4 (3.6 – 4.9)
c	14.6 ± 1.9 (13.7 – 20.3)	18 – 22.3	21.9 ± 2.1 (18.5 – 25.1)	20 ± 2 (18.1 – 23.1)	19.7 ± 2.6 (16.7 – 24.1)
c’	2.3 ± 0.2 (2.0 – 2.8)	–	2.2 ± 0.2 (1.9 – 2.8)	2.2 ± 0.3 (1.9 – 2.6)	1.9 ± 0.2 (1.6 – 2.2)
V	72.2 ± 7.6 (62.4 – 86.9)	73 – 81	76 ± 1 (75 – 78)	75.1 ± 1.9 (71.4 – 77.1)	75.9 ± 1.3 (74 – 78.7)
Stylet length	16.7 ± 0.7 (15.8 – 17.8)	16.5 – 18.0	16.4 ± 0.4 (15.4 – 16.9)	16.3 ± 0.8 (15 – 17)	16.0 ± 0.6 (15 – 17)
Dorsal gland opening from stylet base	3.0 ± 0.5 (2.3 – 3.5)	–	2.9 ± 0.3 (2.4 – 3.1)	–	–
O	17.7 ± 2.9 (13.6 – 22.2)	–	17.4 ± 1.7 (14.3 – 21.3)	–	–
Pharynx length	85 ± 1.0 (82.5 – 85.9)	–	80.1 ± 3.2 (73.1 – 84.2)	89 ± 10.4 (76 – 105)	87 ± 4 (82 – 94)
Pharyngeal overlap	–	–	39.0 ± 7.2 (29.8 – 49.0)	–	
Anterior end to end of pharyngeal gland lobe	101 ± 1.4 (98 – 102)	–	–	125 ± 7.3 (116 – 135)	123 ± 8.3 (113 – 138)
Maximal body diameter	22.7 ± 0.5 (21.6 – 23.1)	–	20.2 ± 2.2 (16.8 – 23.7)	23 ± 3.3 (20 – 29)	22.5 ± 2.1 (19 – 26)
Anal body diameter	13.0 ± 1.2 (10.9 – 14.1)	–	11.2 ± 1.2 (8.9 – 13.2)	12.4 ± 1.5 (11 – 14)	13 ± 0.9 (12 – 14)
Tail length	29.0 ± 0.9 (27.5 – 30.1)	–	24.8 ± 2.3 (21.0 – 27.1)	26.6 ± 2.3 (23 – 29)	24.8 ± 2.3 (23 – 29)
Tail annuli	20 ± 2 (18 – 23)	–	19 ± 2 (16 – 24)	20 ± 2.1 (18 – 23)	19 ± 1.5 (17 – 21)
Phasmid to terminus	14.1 ± 0.4 (13.5 – 14.5)	–	10.6 ± 2.7 (6.0 – 14.9)	–	–

## Description

### Females

Vermiform and slightly curved ventrally after heat-killing and fixation. Labial region continuous from the rest of the body and lip region with three annuli. Under SEM (Figs. 1 C,D), *en face* view showing an oval oral aperture surrounded by six inner labial sensilla, submedian segments fused to oral disc, corresponding to head pattern group 2 according to Corbett and Clark (1983). Stylet was well developed (16–18 μm long) with anteriorly directed rounded knobs. The areolation was only visible at tail level and lateral field with four incisures, with the outer two being entirely crenate, and the inner lines being finely striated. Rounded to oval-shaped metacorpus with short isthmus, pharyngeal gland overlapped ventrally. Excretory pore ws located slightly above pharyngo-intestinal junction. There was a vulva, a transverse slit in ventral view, and well developed post-vulval uterine sac. The tail had (27–30) annuli, subcylindrical, and with rounded to conical, smooth terminus.

### Voucher material

Vouchers (two females) are available in the UGent Nematode Collection (slide UGnem-314) of the Nematology Research Unit, Department of Biology, Ghent University, Ghent, Belgium.

### Molecular characterization

*Pratylenchus delattrei* Four sequences of the D2-D3 28S rDNA (OP646167-OP646170; 571-619 bp; 1–3 bp differences), two ITS rDNA sequences (OP646172-OP646171; 731bp; 17 bp differences) and two *COI* sequences (OP730535-OP7330534; 421 bp; with 100% identical) were generated for *P. delattrei* from Minjar and Adea’a districts (Table 1; Figs. 3 A–C). Based on the D2–D3 sequences, isolates formed the highest supported clade with *P. delattrei* sequences from Cape Verde (KY677820) and two sequences from Iran (JX261949 and JX261948), which are 99.6–100% identical. For ITS, the *P. delattrei* sequences formed a maximally supported clade with three unidentified *Pratylenchus* sp. sequences from India (MN100134, MN100135 and MH375058) with 94–97% similarity (Fig. 3B). Finally, two identical *COI* sequences have been generated for the first time for *P. delattrei*, and these sequences were in a poorly supported sister relationship (0.68 PP) with *P. parazeae* (Fig. 3C).

### Remarks

Male nematodes were not found. As first reported on chickpea and from Ethiopia, this species was recovered from the Minjar and Adea’a districts in the central parts of Ethiopia, both in the rhizosphere and the roots (Table 1). It has been also reported in other African countries, including Madagascar (cotton), Sudan (sugarcane), and Cape Verde (tomato), and from several Asian countries: South Korea, Pakistan, Oman, Iran (on tomato and eggplant, date palm, pigeon pea and peanut, and medicinal plants) (Luc, 1958; Sharma et al., 1992; Jothi et al., 2004; Mani et al., 2005; Castillo & Vovlas, 2007; MajdTaheri et al., 2013; Flis et al., 2018). The studied female morphology and morphometrics are in agreement with the original description (Luc, 1958), and other descriptions of *P. delattrei* from Iran and Cape Verde (MajdTaheri et al., 2013; Flis et al., 2018). The characteristics also agree without variation with the matrix code for the tabular key of Castillo and Vovlas (2007): A2 (three labial annuli), B1 (male absent), C3 (stylet length 16–17 μm), D1 (shape of spermatheca absent or reduced), E2 (V ratio = 75–79.9%), F3 (PUS: 20–24.9 μm), G3 (conoid tail shape), H1 (smooth tail tip), I1 (<30 μm pharyngeal overlapping length), J1 (four lateral field lines), K1 (smooth bands of lateral field structures), and a subcylindrical tail shape with a conical to rounded tail tip. The Ethiopian *P. delattrei* has a slightly longer tail compared to populations from Iran and Cape Verde (27.5–30.1 vs. 21–29 μm); however, the tail length was not included in the original description. In the phylogenetic tree of the ITS region, the Ethiopian *P. delattrei* sequences formed a maximally supported clade with three unidentified *Pratylenchus* species from India; these may therefore also represent *P. delattrei* based on the relatively limited molecular variability (26–49 bp difference). This study links for the first time ITS sequences to *P. delattrei*.

### *Quinisulcius capitatus* (Allen, 1955) Siddiqi, 1971

(Fig. 2)

**Figure 2:**
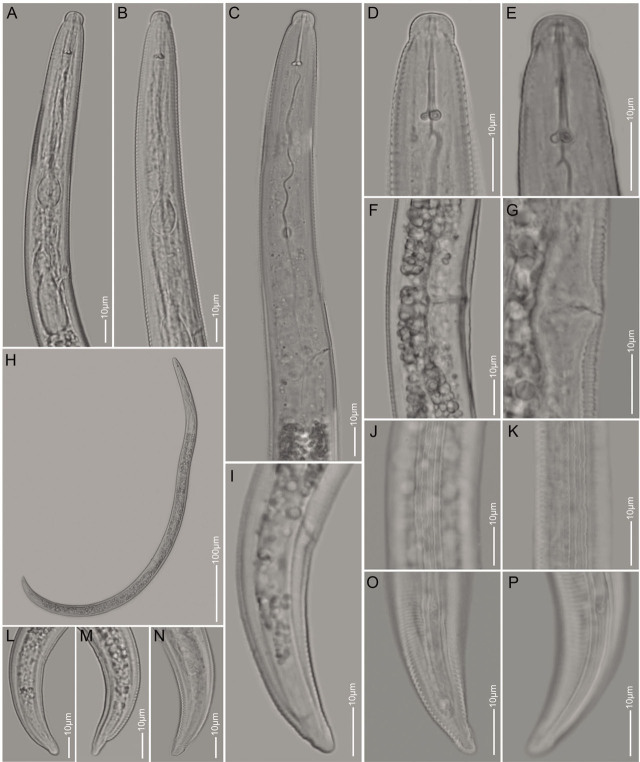
Light microscopy images of *Quinisulcius capitatus*. A–E: Anterior part of the body showing lip and neck regions; F,G: Vulval regions (lateral view); H: Whole female body; J,K: Lateral field showing five distinct incisures; I, L–P: Tail region lateral view.

### Measurements

See Table 3.

**Table 3.** Comparison of important morphological character and morphometrics of the Ethiopian *Quinisulcius capitatus*, found from chickpea in Ethiopia, with original description from USA (Allen, 1955), and two other *Q. capitatus* from Ethiopia and Canada. All measurements are in μm and in the form: mean ± s.d. (range).

**Character**	***Q. capitatus* from chickpea in Ethiopia (present study)**	***Q. capitatus* from pear in USA (Allen, 1955)**	***Q. capitatus* from coffee in Ethiopia (Mekete et al., 2008)**	“Q. capitatus”^*^ from grass in Canada (Munawar et al., 2021)
n	10	13	10	20
L	699 ± 11.6 (667 – 707)	630 – 850	630 – 790	810.3 ± 44.6 (744.0 – 911.0)
a	31.2 ± 1.3 (29.7 – 34.4)	30 – 38	30.9 – 38.6	41.4 ± 1.8 (38.6 – 43.7)
b	5.4 ± 0.2 (5.1 – 5.7)	5.0 – 5.8	–	5.5 ± 0.3 (5.0 – 6.3)
c	14.7 ± 0.7 (14.1 – 16.3)	12 – 17	15.3 – 17.6	22.4 ± 1.1 (19.9 – 23.8)
c’	1.9 ± 0.2 (1.5 – 2.1)	–	–	2.6 ± 0.2 (2.2 – 3.2)
V	56.6 ± 2.2 (52.7 – 59.0)	51 – 58	54.7 – 63.6	57.4 ± 1.5 (53.4 – 59.8)
Stylet length	18.8 ± 0.6 (17.8 – 19.7)	16 – 18	15 – 18	18.3 ± 1.0 (15.5 – 20.4)
Lip height	4.0 ± 0.5 (3.2 – 4.5)	–	–	4.0 ± 0.2 (3.7 – 4.4)
Lip width	7.6 ± 0.5 (7.0 – 8.3)	–	–	7.6 ± 0.4 (6.9 – 8.3)
Median bulb length	16.1 ± 0.3 (15.7 – 16.6)	–	–	14.0 ± 1.6 (11.3 – 16.9)
Median bulb width	13.1 ± 0.6 (12.2 – 13.9)	–	–	10.4 ± 1.4 8.4 – 14.2
Pharyngeal length	129 ± 5.1 (123 – 138)	–	–	147.8 ± 5.8 (140.2 – 159.0)
SE pore from anterior end	121 ± 3.3 (113 – 123)	–	121	128.6 ± 5.3 (121.0 – 139.0)
Midbody diameter	22.4 ± 1.0 (20.5 – 23.5)	21 – 27	–	–
Tail length	47.8 ± 3.0 (41.0 – 50.0)	–	–	35.8 ± 2.4 (31.3 – 40.4)
Anal body diameter	25.3 ± 3.2 (23.0 – 34.2)	–	–	–
Phasmid position	Middle of tail	Middle of tail	–	Middle of tail

* *Q. capitatus* Canada is likely misidentified, see remarks.

### Description

#### Females

The body of females spiral, or become C shaped after heat relaxation. The lip region hemispherical, set off with necks, with having five to six annulations, strong stylet (17.8–19.7 μm), long, rounded basal knobs, lateral field with five incisures. Rounded median bulb with strongly developed central valves, slender isthmus surrounded by nerve ring and conspicuous rounded cardia. Deirids absent and excretory pores at level between anterior margin and the middle of the basal pharyngeal bulb. Protruding vulva lips and poorly developed round spermatheca. The tail terminus conoid, distinctly annulated, tail cylindrical, with distinct phasmid at the middle of the tail.

#### Male

Not found

#### Molecular characterization

*Quinisulcius capitatus* Three identical D2–D3 of 28S (OP62631–OP626321; 490–693 bp), three identical ITS rDNA (OP646173–OP646175; 882–915 bp), and three identical *COI* gene (OP627909–OP627911; 350 bp) sequences were generated (Table 1; Figs. 4A–C). The D2–D3 sequences formed a maximum supported clade with nine 99–100% identical *Q. capitatus* sequences from Pakistan (MT703017–MT703025) (Fig. 4A). Our ITS rDNA sequences also formed a maximally supported clade with seven identical *Q. capitatus* ITS sequences from Pakistan (MT703005–MT703011) (Fig. 4B). However, two *Q. capitatus* sequences from Canada (MW027537–MW027538) are only 83% similar and were in an unresolved position with *Tylenchorhynchus* and *Q. curvus* sequences (Fig. 4B). The three identical *COI* sequences are the first sequences for *Q. capitatus*, and these sequences showed a weakly supported sister relationship with *Amplimerlinius icarus* and *Tylenchorhynchus* (0.63 vs. 0.58 PP) (Fig. 4C).

**Figure 3:**
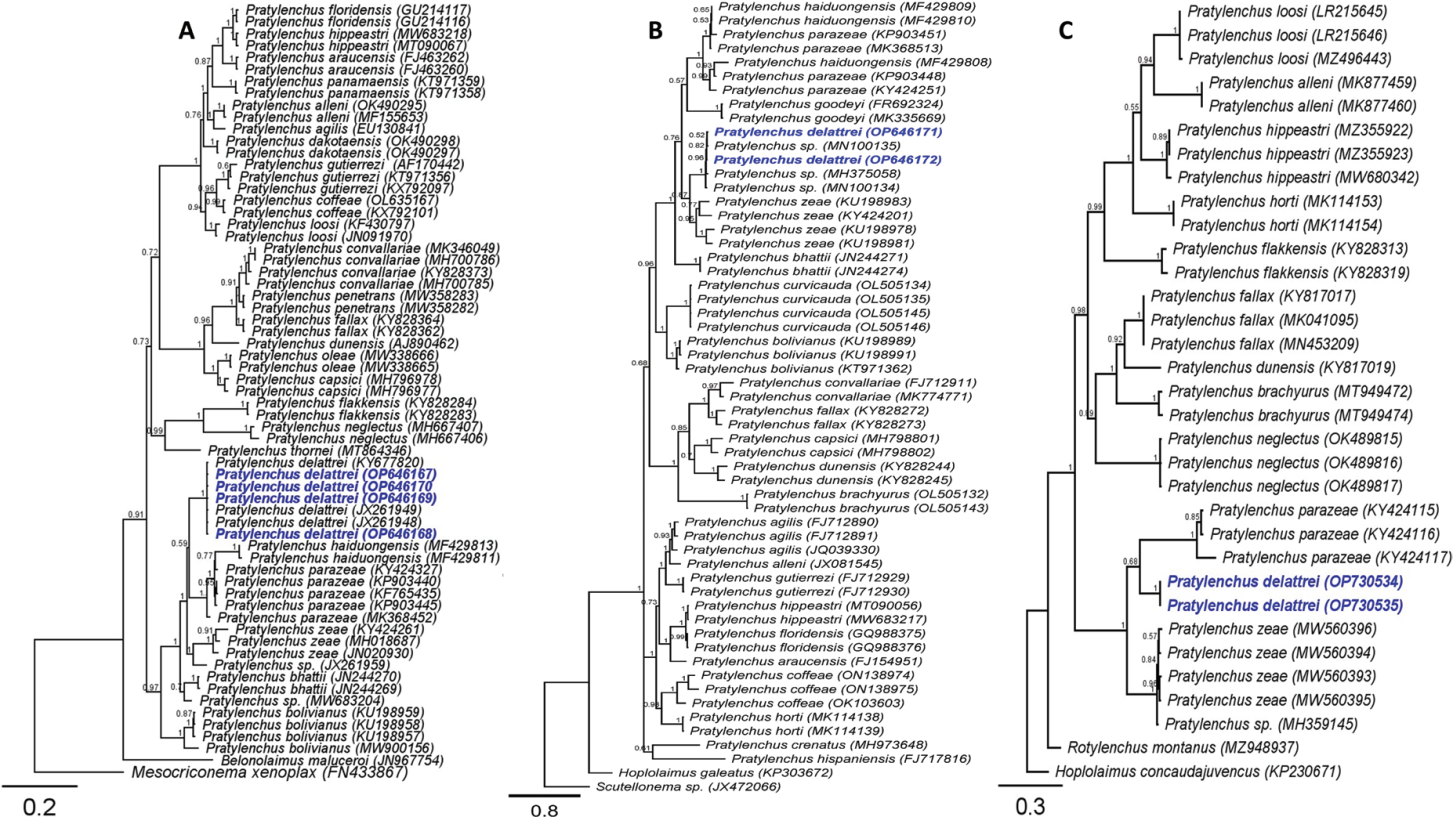
Bayesian 50% majority rule consensus phylogeny of *Pratylenchus delattrei* from Ethiopia and related species based on 28S (A) and (B) ITS of rDNA genes and (C) *COI* of mtDNA using a GTR model. Branch support is indicated with PP. The sequences from this study were marked by blue color and bold font.

### Remarks

**Figure 4:**
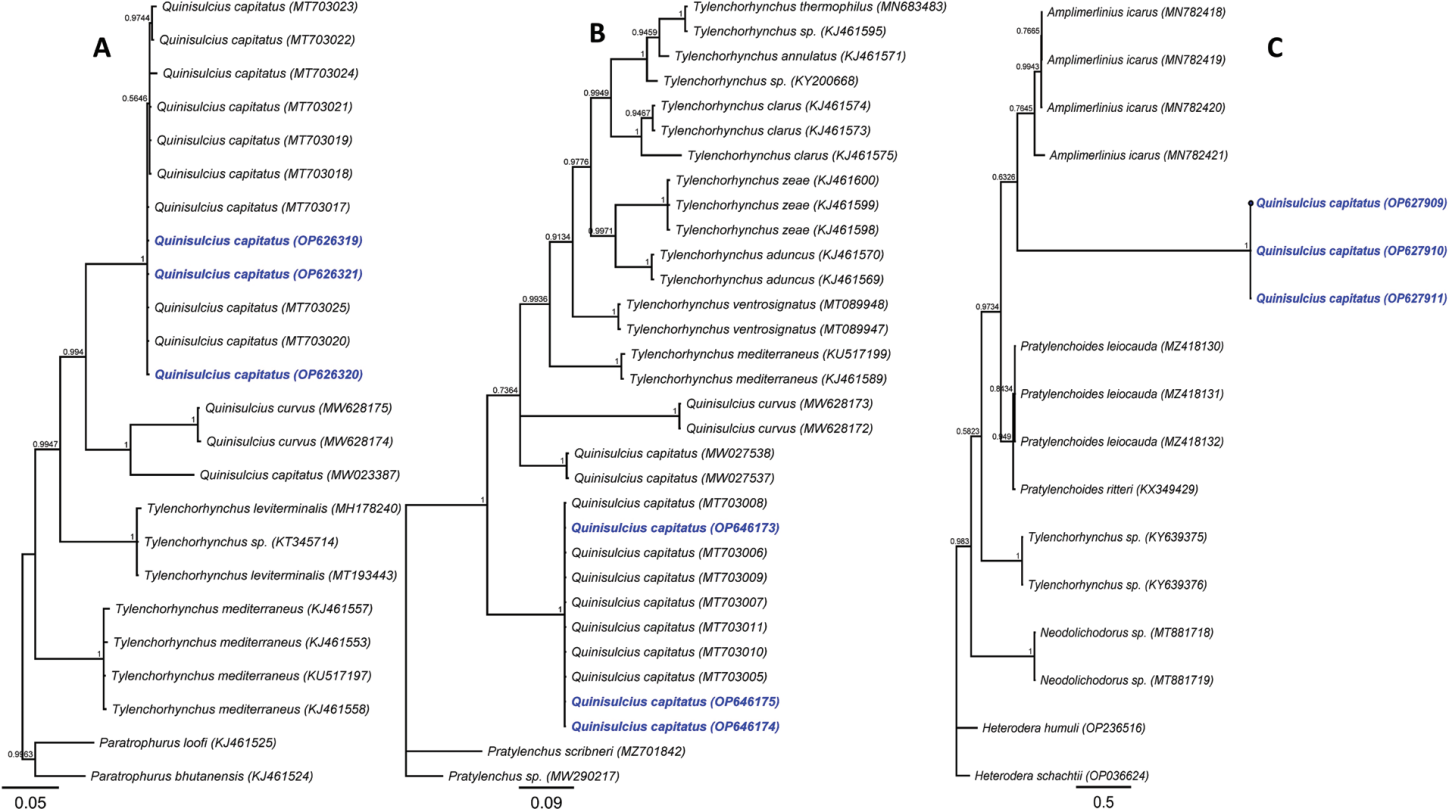
Bayesian 50% majority rule consensus phylogeny of *Quinisulcius capitatus* from Ethiopia and related species on 28S (A) and (B) ITS of rDNA genes and (C) *COI* of mtDNA using a GTR model. Branch support is indicated with PP. The sequences from this study were marked by blue color and bold font.

The studied specimens are morphologically and morphometrically similar to the original description (Allen, 1955) and subsequent descriptions by Mekete et al. (2008), Munawar et al. (2021), and Iqbal et al. (2021), except for the slightly longer stylet compared to populations from coffee in Ethiopia (17.8–19.7 vs. 15–18 μm) and longer tail compared to the Canadian population (41.0–50.0 vs. 31.3–40.4 μm) (Table 3). All of the specimens have five incisures in the lateral field and also conoid, enlarged and striated terminus shape (Fig. 2), agreeing with genus *Quinisulcius*, which sets it apart from *Tylenchorhynchus* (5 vs. 3–4) according to the key of Hunt et al. (2012). As in the current study, males have rarely been found (Hopper, 1959; Siddiqi, 1971; Knobloch and Laughlin, 1973; Maqbool, 1982; Mekete et al., 2008; Geraert, 2011). However, Iqbal et al. (2021), described *Q. capitatus* male specimens from apple, tomato, maize, potato, cabbage, and onion in Pakistan. *Quinisulcius capitatus* is known to parasitize over 27 plants across all continents (North America, Central and South America, temperate parts of Europe, Africa, Asia, Australia, and New Zealand) (Munawar et al., 2021). In Africa, this species has been reported in Ethiopia from coffee (Mekete et al., 2008), soybean in South Africa (Mbatyoti et al., 2020), and tomato and carrot in Benin (Baimey et al., 2009). The Ethiopian *Q. capitatus* specimens formed a well-supported clade with the Pakistan populations in our D2–D3 of 28S and ITS rDNA, however, the tree topology to the Canadian *Q. capitatus* population was not resolved for both gene regions (Figs. 4A,B). This suggests that the Canadian populations may have been mislabelled.

## Discussion

Using morphological and molecular data, *P. delattrei* was detected for the first time in chickpea, and for the first time in Ethiopia. Other RLN species, i.e., *P. zeae, P. alleni, P. alkan, P. erzurumensis P. mulchandi, P. coffeae, P. thornei, P. neglectus, P. mediterraneus, P. penetrans, P. brachyurus*, and *P. minyus,* have previously been reported from the root and rhizosphere of chickpea, and their associated damage to crops has been widely studied in different countries (Di Vito et al., 1992; Di Vito et al., 1994, Castillo et al., 1996; Ali and Sharma, 2003; Castillo et al., 2008; Hollaway et al., 2008; Thompson et al., 2010; Sikora et al., 2018; Zwart et al., 2019; Behmand et al., 2022; Rostad et al., 2022). Accurate identification of RLN species is important in applying appropriate pest management strategies, so it is remarkable that despite the status of chickpea as an important leguminous crop, neither the presence nor the damage potential of *Pratylenchus* spp. have been studied in Ethiopia. Furthermore, although *Pratylenchus* contains over 100 species (Janssen et al., 2017b; Singh et al., 2018; Nguyen et al., 2019; Handoo et al., 2021), only four (*P. zeae*, *P. brachyurus*, *P. coffeae* and *P. goodeyi*) have so far been reported in Ethiopia (Peregrine and Bridge, 1992; Abebe et al., 2015). Similarly, the genus *Quinisulcius* contains over 17 species and can multiply in several host plants (Geraert, 2011; Hussain et al., 2019; Iqbal et al., 2021; Munawar et al., 2021), including chickpea (Di Vito et al., 1994; Ali and Sharma, 2003; Catillo et al., 2008), yet none of the *Quinisulcius* species have ever been reported from chickpea in Ethiopia. It is therefore striking that this current study has generated not only the first *COI* sequences of *Q. capitatus* and *P. delattrei*, but also the very first sequences of the genus *Quinisulcius*. Although mitochondrial genes, and especially *COI*, appear to be very informative for nematode diagnostics (Singh et al., 2021; Nguyen et al., 2022), nematodes remain one of the animal taxa with the lowest representation in the *COI* barcode database as compared to rDNA gene markers, according to a GenBank search conducted by Thomas et al. (2017). Instead, 18S and 28S ribosomal sequences have been traditionally the focus for nematode barcoding (Blaxter et al., 1998; Powers et al., 2021), although the mitochondrial *COI* gene is the designated marker for many animals since it is present in multiple copies per cell (Powers et al., 2021).

The first reports of both species and their morphological and molecular characterizations presented in the current study form a solid basis for future research on their economic impact, their interactions with other pathogens, and the development of nematode management strategies for Ethiopian chickpea growers. It is clear that the distribution and effect of these two species in other leguminous crops in Ethiopia, as well as in other SSA countries, represent important subjects for future investigation.

## References

[j_jofnem-2023-0027_ref_001] Abebe E., Tesfamariam M., Awol S., Meressa B. H., Mesfin W., Temesgen A., Gezahegn G., Birhan A. (2015). Research on plant-parasitic and entomopathogenic nematodes in Ethiopia: a review of current state and future direction. Nematology.

[j_jofnem-2023-0027_ref_002] Ali S. S., Sharma S. B. (2003). Nematode survey of chickpea production areas in Rajasthan, India. Nematologia Mediterranea.

[j_jofnem-2023-0027_ref_003] Allen M. W. (1955). A review of the genus *Tylenchorhynchus*. University of California Publications in Zoology.

[j_jofnem-2023-0027_ref_004] Bafokuzara N. D. (1996). Incidence of different nematodes on vegetable and fruit crops and preliminary assessment of yield loss due to *Meloidogyne* species in Uganda. Nematology Brasil.

[j_jofnem-2023-0027_ref_005] Baimey H., Coyne D., Dagnenonbakin G., James B. (2009). Plant-parasitic nematodes associated with vegetable crops in Benin: Relationship with soil physico-chemical properties. Nematologia Mediterranean.

[j_jofnem-2023-0027_ref_006] Barker K. R., Nusbaum C. J., Nelson L. A. (1969). Effects of storage temperature and extraction procedure on recovery of plant-parasitic nematodes from field soils. Journal of Nematology.

[j_jofnem-2023-0027_ref_007] Behmand T., Berger J., Elekcioglu I. H., Aydogan A., Kahraman A. (2022). Population fluctuations and reaction of chickpea genotypes to root lesion nematodes (*Pratylenchus thornei* and *P. neglectus*) in Turkey. Indian Phytopathology.

[j_jofnem-2023-0027_ref_008] Behmand T., Elekcioğlu N. Z., Berger J., Can C., Elekcioğlu H. (2018). Determination of plant parasitic nematodes associated with chickpea in Turkey. Turkish Journals of Entomology.

[j_jofnem-2023-0027_ref_009] Blaxter M. L., De Ley P., Garey J. R., Liu L. X., Scheldeman P., Vierstraete A., Vanfleteren J. R., Mackey L. Y., Dorris M., Frisse L. M., Vida J. T., Thomas W. K. (1998). A molecular evolutionary framework for the phylum Nematoda. Nature.

[j_jofnem-2023-0027_ref_010] Bowles J., Blair D., McManus D. P. (1992). Genetic variants within the genus *Echinococcus* identified by mitochondrial DNA sequencing. Molecular and Biochemical Parasitology.

[j_jofnem-2023-0027_ref_011] Castillo P., Gomez-Barcina A., Jiménez-Díaz R. M. (1996). Plant-parasitic nematodes associated with chickpea in southern Spain and effect of soil temperature on reproduction of *Pratylenchus thornei*. Nematologica.

[j_jofnem-2023-0027_ref_012] Castillo P., Vovlas N. (2007). *Pratylenchus* (*Nematoda*: *Pratylenchidae*): Diagnosis, Biology, *Pathogenicity* and *Management*.

[j_jofnem-2023-0027_ref_013] Castillo P., Navas-Cortés J. A., Landa B. B., Jiménez-Díaz R. M., Vovlas N. (2008). Plant-parasitic nematodes attacking chickpea and their inplanta interactions with rhizobia and phytopathogenic fungi. Plant Disease.

[j_jofnem-2023-0027_ref_014] Corbett D. C. M., Clark S. A. (1983). Surface features in the taxonomy of *Pratylenchus* species. Review de Nematology.

[j_jofnem-2023-0027_ref_015] Coye D. L., Cortada L., Dalzell J. J., Claudius-Cole A. O., Solveig H., Luambano N., Talwana H. (2018). Plant-parasitic nematodes and food security in sub-Saharan Africa. Annual Review of Phytopathology.

[j_jofnem-2023-0027_ref_016] Dadi L., Regassa S., Fikre A., Mitiku D. (2005). Adoption of chickpea varieties in the central highlands of Ethiopia. Ethiopian Agricultural Research Organization.

[j_jofnem-2023-0027_ref_017] Derycke S., Vanaverbeke J., Rigaux A., Backeljau T., Moens T. (2010). Exploring the use of cytochrome oxidase *c* subunit 1 (*COI*) for DNA barcoding of free-living marine nematodes. PLoS ONE.

[j_jofnem-2023-0027_ref_018] Di Vito M., Greco N., Saxena M. C. (1992). Pathogenicity of *Pratylenchus thornei* on chickpea in Syria. Nematologica Mediterranea.

[j_jofnem-2023-0027_ref_019] Di Vito M., Greco N., Halila H. M., Mabsoute L., Labdi M., Beniwal S. P. S., Saxena M., Singh K. B., Solh M. B. (1994). Nematodes of cool season food legumes in North Africa. Nematologica Mediterreanea.

[j_jofnem-2023-0027_ref_020] FAOSTAT (Food and Agriculture Organization of United Nations) (2020). Dry Bean.

[j_jofnem-2023-0027_ref_021] Fikre A., Desmae H., Ahmed S. (2020). Tapping the economic potential of chickpea in sub-Saharan Africa. Agronomy.

[j_jofnem-2023-0027_ref_022] Flis L., Dobosz R., Rybarczyk-Mydłowska K., Wasilewska-Nascimento B., Kubicz M., Winiszewska G. (2018). First report of the lesion nematodes: *Pratylenchus brachyurus* and *Pratylenchus delattrei* on tomato (*Solanum lycopersicum* L.) plants in Cape Verde. Helminthologia.

[j_jofnem-2023-0027_ref_023] Geraert E. (2011). The Dolichodoridae of the world. Identification of the family Dolichodoridae (Nematoda).

[j_jofnem-2023-0027_ref_024] Greco N., Di Vito M., Saxena M. C. (1992). Plant parasitic nematodes of cool season food legumes in Syria. Nematologia Mediterranea.

[j_jofnem-2023-0027_ref_025] Handoo Z. A., Yan G., Kantor M. R., Huang D., Chowdhury I. A., Plaisance A., Bauchan G. R., Mowery J. D. (2021). Morphological and Molecular Characterization of *Pratylenchus dakotaensis* n. sp. (Nematoda: Pratylenchidae), a New Root-Lesion Nematode Species on Soybean in North Dakota, USA. Plants.

[j_jofnem-2023-0027_ref_026] Hollaway G. J., Vanstone V. A., Nobbs J., Smith J. G., Brown J. S. (2008). Pathogenic nematodes of cereal crops in south-west Victoria, Australia. Australia Plant Pathology.

[j_jofnem-2023-0027_ref_027] Hooper D., Hallmann J., Subbotin S., Luc M., Sikora R. A., Bridge J. (2005). Extraction, processing and detection of plant and soil nematodes. Plant parasitic nematodes in subtropical and tropical agriculture.

[j_jofnem-2023-0027_ref_028] Hopper B. E. (1959). Three new species of the genus *Tylenchorhynchus* (Nematoda: Tylenchida). Nematology.

[j_jofnem-2023-0027_ref_029] Huelsenbeck J. P., Ronquist F. (2001). MRBAYES: Bayesian inference of phylogenetic trees. Bioinformatics.

[j_jofnem-2023-0027_ref_030] Hunt D. J., Bert W., Siddiqi M. R., Manzanilla-López R. H., Marbán-Mendoza N. (2012). Tylenchidae and Dolichodoridae. Practical Plant Nematology.

[j_jofnem-2023-0027_ref_031] Hussain S., Erum Y. I., Shahina F. (2019). Compendium of the genus *Quinisulcius* and observations on occurrence of *Q. capitatus*. Pakistan Journal of Zoology.

[j_jofnem-2023-0027_ref_032] Iqbal E., Shokoohi E., Hussain S., Mashela P. W., Kazi N. (2021). Morphological and molecular characterization of plant parasitic nematode *Quinisulcius capitatus* (Allen, 1955) Siddiqi, 1971 from Gilgit Province, Pakistan. European Journal of Plant Pathology.

[j_jofnem-2023-0027_ref_033] Janssen T., Karssen G., Orlando V., Subbotin S.A., Bert W. (2017a). Molecular characterization and species delimiting of plant-parasitic nematodes of the genus *Pratylenchus* from the *penetrans* group (Nematoda: Pratylenchidae). Molecular Phylogenetics and Evolution.

[j_jofnem-2023-0027_ref_034] Janssen T., Karssen G., Couvreur M., Waeyenberge L., Bert W. (2017b). The pitfalls of molecular species identification: a case study within the genus *Pratylenchus* (Nematoda: Pratylenchidae). Nematology.

[j_jofnem-2023-0027_ref_035] Jones J. T., Haegeman A., Danchin E. G. J., Gaur H. S., Helder J., Jones M. G. K., Kikuchi T., Manzanilla-Lopez R., Palomares-Rius J., Wesemael W. M. L., Roland N. P. (2013). Top 10 plant-parasitic nematodes in molecular plant pathology. Molecular Plant Pathology.

[j_jofnem-2023-0027_ref_036] Jothi G., Babu R. S., Ramakrishnan S., Rajendran G. (2004). Management of root lesion nematode, *Pratylenchus delattrei* in cros-sandra using oil cakes. Bioresource Technology.

[j_jofnem-2023-0027_ref_037] Knobloch N. A., Laughin C. W. (1973). A collection of plant parasitic nematodes (Nematoda) from Mexico with description of three new species. Nematology.

[j_jofnem-2023-0027_ref_038] MajdTaheri Z., Tanha Maafi Z., Subbotin S. A., Pourjam E., Eskandari A. (2013). Molecular and phylogenetic studies on Pratylenchidae from Iran with additional data on *Pratylenchus delattrei*, *Pratylenchoides alkani* and two unknown species of *Hirschmanniella* and *Pratylenchus*. Nematology.

[j_jofnem-2023-0027_ref_039] Mani A., Handoo Z. A., Livingstone S. (2005). Plant parasitic nematodes associated with date palm trees (*Phoenix dactylifera*) in the Sultane in Oman. Nematropica.

[j_jofnem-2023-0027_ref_040] Maqbool M. A. (1982). Description of *Quinisulcius solani* n. sp., (Nematoda: Tylenchorhynchidae) with a key to the species and data on *Scutylenchus koreanus* from Pakistan. Journal of Nematology.

[j_jofnem-2023-0027_ref_041] Mbatyoti A., Daneel M. S., Swart A., Marais M., De Waele D., Fourie H. (2020). Plant-parasitic nematode assemblages associated with glyphosate tolerant and conventional soybean cultivars in South Africa. African Zoology.

[j_jofnem-2023-0027_ref_042] Mekete T., Sikora R. A., Kiewnick S., Hallmann J. (2008). Description of plant parasitic nematodes associated with coffee in Ethiopia. Nematologica Mediterranea.

[j_jofnem-2023-0027_ref_043] Munawar M., Yevtushenko D. P., Castillo P. (2021). Integrative taxonomy, distribution, and host associations of *Geocenamus brevidens* and *Quinisulcius capitatus* from southern Alberta, Canada. Journal of Nematology.

[j_jofnem-2023-0027_ref_044] Nguyen H. T., Nguyen T. D., Le T. M. L., Trinh Q. P., Bert W. (2022). Remarks on phylogeny and molecular variations of criconematid species (Nematoda: Criconematidae) with case studies from Vietnam. Scientific Report.

[j_jofnem-2023-0027_ref_045] Nguyen H. T., Trinh Q. P., Couvreur M., Singh R. P., Decraemer W., Bert W. (2019). Molecular and morphological characterisation of a new root-lesion nematode, *Pratylenchus horti* n. sp. (Tylenchomorpha: *Pratylenchidae*), from Ghent University Botanical Garden. Nematology.

[j_jofnem-2023-0027_ref_046] Peregrine W. T. H., Bridge J. (1992). The lesion nematode, *Pratylenchus goodeyi*, an important pest of enset in Ethiopia. Tropical Pest Management.

[j_jofnem-2023-0027_ref_047] Powers T., Harris T., Higgins R., Mullin P., Sutton L., Powers K. (2011). MOTUs, morphology, and biodiversity estimation: a case study using nematodes of the suborder Criconematina and a conserved 18S DNA barcode. Journal of Nematology.

[j_jofnem-2023-0027_ref_048] Powers T. O., Harris T. S., Higgins R. S., Mullin P. G., Powers K. S. (2021). Nematode biodiversity assessments need vouchered databases: A BOLD reference library for plant-parasitic nematodes in the superfamily Criconematoidea. Genome.

[j_jofnem-2023-0027_ref_049] Reen R. A., Mumford M. H., Thompson J. P. (2019). Novel sources of resistance to root-lesion nematode (*Pratylenchus thornei*) in a new collection of wild *Cicer* species (*C. reticulatum* and *C. echinospermum*) to improve resistance in cultivated chickpea. Phytopathology.

[j_jofnem-2023-0027_ref_050] Riley I. T., Kelly S. J. (2002). Endo parasitic nematodes in cropping soils of Western Australia. Australian Journal of Experimental Agriculture.

[j_jofnem-2023-0027_ref_051] Rostad E., Reen A., Humford H., Zwart S., Thompson P. (2022). Resistance to root-lesion nematode *Pratylenchus neglectus* identified in a new collection of two wild chickpea species (*Cicer reticulatum* and *C. echinospermum*) from Turkey. Plant Pathology.

[j_jofnem-2023-0027_ref_052] Seinhorst J. W. (1959). A rapid method for the transfer of nematodes from fixative to anhydrous glycerin. Nematologica.

[j_jofnem-2023-0027_ref_053] Sharma S. B., Smith D. H., McDonald D. (1992). Nematode constraints of chickpea and pigeon pea production in the semiarid tropics. Plant Disease.

[j_jofnem-2023-0027_ref_054] Shiferaw B., Jones R., Silim S., Tekelewold H., Gwata E. (2007). Analysis of production costs, market opportunities and competitiveness of Desi and Kabuli chickpeas in Ethiopia.

[j_jofnem-2023-0027_ref_055] Siddiqi M. R. (1971). Structure of the oesophagus in the classification of the superfamily Tylenchoidea (Nematoda). Indian Journal of Nematology.

[j_jofnem-2023-0027_ref_056] Sikora R. A., Claudius-Cole B., Sikora E. J., Sikora R. A., Coyne D., Hallman J., Timper P. (2018). Nematode parasites of food legumes. Plant parasitic nematodes in subtropical and tropical agriculture.

[j_jofnem-2023-0027_ref_057] Singh P. R., Couvreur M., Decraemer W., Bert W. (2019). Survey of slug-parasitic nematodes in East and West Flanders, Belgium, and description of *Angiostoma gandavensis* n. sp. (Nematoda: Angiostomidae) from arionid slugs. Journal of Helminthology.

[j_jofnem-2023-0027_ref_058] Singh P. R., Karssen G., Couvreur M., Subbotin S. A., Bert W. (2021). Integrative taxonomy and molecular phylogeny of the plant-parasitic nematode genus paratylenchus (Nematoda: Paratylenchinae): linking species with molecular barcodes. Plants.

[j_jofnem-2023-0027_ref_059] Singh P. R., Nyiragatare A., Janssen T., Couvreur M., Decraemer W., Bert W. (2018). Morphological and molecular characterisation of *Pratylenchus rwandae* n. sp. (Tylenchida: *Pratylenchidae*) associated with maize in Rwanda. Nematology.

[j_jofnem-2023-0027_ref_060] Singh R., Sharma P., Varshney R. K., Sharma S. K., Singh N. K. (2008). Chickpea improvement: Role of wild species and genetic markers. Biotechnology and Genetic Engineering Review.

[j_jofnem-2023-0027_ref_061] Subbotin S. A., Sturhan D., Chizhov V. N., Vovlas N., Baldwin J. G. (2006). Phylogenetic analysis of Tylenchida Thorne, 1949 as inferred from D2 and D3 expansion fragments of the 28S rRNA gene sequences. Nematology.

[j_jofnem-2023-0027_ref_062] Thomas P., Steiner F. M., Schlick-Steiner B. C., Arthofer W. (2017). Are we ready to detect nematode diversity by next generation sequencing?. Ecology and Evolution.

[j_jofnem-2023-0027_ref_063] Thompson J. P., Clewett T. G., Sheedy J. G. (2010). Occurrence of root-lesion nematodes (*Pratylenchus thornei* and *P. neglectus*) and stunt nematode (*Merlinius brevidens*) in the northern grain region of Australia. Australasian Plant Pathology.

[j_jofnem-2023-0027_ref_064] Upadhaya A., Yan G., Pasche J., Kalil A. (2018). Occurrence and distribution of vermiform plant–parasitic nematodes and the relationship with soil factors in field pea (*Pisum sativum*) in North Dakota, USA. Nematology.

[j_jofnem-2023-0027_ref_065] Vrain T. C., Wakarchuk D. A., Levesque A. C., Hamilton R. I. (1992). Intraspecific rDNA restriction fragment length polymorphism in the *Xiphinema americanum* group. Fundamental and Applied Nematology.

[j_jofnem-2023-0027_ref_066] Zwart R. S., Thudi M., Channale S., Manchikatla P. K., Varshney R. K., Thompson J. P. (2019). Resistance to plant-parasitic nematodes in chickpea: current status and future perspectives. Frontiers in Plant Science.

